# Clinical Evaluation of the Nasopalatine Canal in Implant-Prosthetic Treatment: A Pilot Study

**DOI:** 10.3390/dj8020030

**Published:** 2020-04-01

**Authors:** Enrique Fernández Bodereau, Viviana Yolanda Flores, Pablo Naldini, Daniel Torassa, Patricia Tortolini

**Affiliations:** 1 Department of Fixed Prosthodontics, Oral Surgery and Implantology, Faculty of Dentistry, Universidad Nacional de Córdoba, Córdoba 5000, Argentina; pnaldini@hotmail.com (P.N.); dtorassa@unc.edu.ar (D.T.); Ptortolini@gmail.com (P.T.); 2 Department of Oral Biology, Faculty of Dentistry, Universidad Nacional de Córdoba, Córdoba 5000, Argentina; vivyflores@gmail.com

**Keywords:** nasopalatine canal, dental implant, block grafting, guided bone regeneration

## Abstract

Implant-prosthetic rehabilitation of missing teeth in the anterior maxilla is often challenging due to ongoing bone resorption and remodeling events and may require regeneration procedures involving the nasopalatine canal (NPC). We describe a surgical approach with a block graft in relation to the NPC and evaluate clinical performance, sensory perception, and aesthetic result of the implant-prosthetic treatment over a two- to nine-year (mean: 3.5 years) follow-up. Ten implants (six in the right central incisor and four in the left central incisor) were, respectively, placed in 10 consecutive patients with bone defects affecting the NPC and unfavorable widening of the incisive foramen. Treatment stages included: (1) Diagnosis: evaluation of clinical-aesthetic parameters using Cone Beam Computed Tomography; (2) Surgery: block graft placement by palatine and incisal with simultaneous guided bone regeneration, and late (6–10 months) implant placement; (3) Prosthetics: placement of a screw-retained crown (torque of 32 N/cm). At treatment initiation, all the NPCs evaluated in our study were free of pathologies. Treatment evaluation included bone crest thickness, neurosensory status, patients’ treatment perception, and pink and white aesthetic scores (PES/WES). Pre-surgery, anterior ridge thickness at the level of the incisive foramen was (mean ± SD) 3.5 ± 2 mm, 5.4 ± 1.5 mm, and 6.1 ± 1.9 mm at heights of 4, 8, and 14 mm apical to the marginal bone crest, respectively. Post-treatment values were, respectively, 10.1 ± 2.0 mm, 10.5 ± 1.0 mm, and 13.4 ± 3.0 mm. The perception of treatment with the aesthetic pink and white indices (PES/WES) was an average of 7.5 and 7 points, respectively, out of a total of 10 each index, with a recovery of 100% of the neurosensory perception of the area. We propose that bone augmentation using block and particulate graft material can compensate for anatomical variations in the NPC, optimize implant’s three-dimensional positioning and improve facial contour, providing tissue and implant stability and good aesthetic outcomes.

## 1. Introduction

Premature tooth loss is one of the main causes of bone loss. The rehabilitation of the toothless gap must allow the implant-prosthetic restoration to be surrounded by bone and soft tissue in harmony with the existing dentition [1]. The implant specialist must evaluate each clinical case, classifying it as simple, advanced, or complex (SAC) [2] Assessing the degree of complexity and the surgical and aesthetic risks of the prosthetic rehabilitation will aid to achieve predictable results [3].

The nasopalatine canal (NPC; also known as the incisive canal or anterior palatal canal) is found at the medial margin of the anterior palatal processes of the maxillary bones and communicates the roof of the oral cavity with the floor of the nasal cavity [4,5]. The NPC opens into the palatal vault through the incisor foramen, which is covered by the incisor papilla, and in most cases, it forks into two Y-shaped conduits that open on either side of the nasal septum (Stenson’s foramina) [6]. The NPC is traversed by the nasopalatine nerve and the medial sphenopalatine vessels, in association with adipose tissue and minor salivary glands [7]. In addition to the Y-shaped conformation described above (referred to as type C), which itself presents several variations, single canal (type A) and double canal (type B) variants are also common [4,8]. During surgical planning, attention must also be paid to rare NPC variations, including trifurcation [9] and the presence of an additional NPC [10], as well as to potential the presence of nasopalatine cysts, which show a prevalence of ~1% in the general adult population [11].

Considerable reabsorption of the vestibular and palatal tables may occur over time after tooth loss in the anterior maxilla. Hence, during prosthetic rehabilitation, the stability of the fixation may be compromised and regeneration involving the NPC must be carried out [9,12]. The residual ridge and its relationship with the NPC are analyzed through three-dimensional images obtained by Cone Beam Computed Tomography (CBCT) [13,14], which allows pinpointing anatomical reference points for detailed evaluation. In this way, damage to the neurovascular bundle, sensory complications, and/or failure of osseointegration can be avoided [1,15].

In order to establish an adequate bone structure containing the implant and a correct position for rehabilitation of the missing tooth, bone augmentation using particulate bone grafting and guided bone regeneration (GBR), block grafting (with or without GBR), ridge corticotomy, or osteogenic distraction may be approached [8,16,17]. Extra-oral bone grafts, especially of the iliac crest, have been used since 1980 for the reconstruction of bone defects in implant rehabilitation. Currently, bone grafts obtained from intraoral sites are successfully used [18,19].

The classification of defects for hard and soft tissues in the anterior single-unit gap described by Palacci et al. [20] is considered relevant since this classification pattern is a guide for surgical practice in the area of implantology. Likewise, the protocol of implant placement and loading of Gallucci et al. [21] may also serve as a guide. Detailed knowledge of the variations in the shape, number, and size of the NPC is fundamental to surgical procedures in order to prevent damage to nerves and arteries.

To date, only a few reports addressed NCP enucleation prior to implant placement to restore lost teeth in the anterior maxilla in cases with no pre-existing NPC pathology [22,23]. Therefore, the objective of this study was to describe a surgical approach involving removal of the NPC’s neurovascular bundle and block grafting prior to prosthetic restoration of central incisors, and to evaluate clinical performance, aesthetic results, and patients’ sensory perception and overall treatment satisfaction.

## 2. Materials and Methods

A clinical study was performed on a series of 10 consecutive patients, 20 years of age and older, with an anterosuperior toothless gap at the level of the right or left central incisors (1.1 or 2.1), to place an implant-supported fixed prosthesis with follow-up between 2 and 9 years (mean: 3.5 years). All patients spontaneously consulted the Specialty of Fixed, Removable and Implantology of the Faculty of Dentistry, National University of Córdoba, Argentina, between February 2008 and December 2017. All subjects gave their informed consent for inclusion before they participated in the study. The study was carried out in accordance with the revised Declaration of Helsinki (2013) on clinical research in humans and was approved by the Institutional Committee on Ethics in Health Research (ODO CAI-CIEIS N° 16I in Act 114) of National University of Córdoba on 23 October 2017. The inclusion criteria were patients with vertical or horizontal bone defects or a combination of both, mainly affecting the palatal area, a bone defect that includes the NPC with a wide incisor foramen; partial edentulism for at least one year; absence of significant medical conditions; and patients over 20 years of age.

The exclusion criteria were (1) systemic disease that could compromise wound healing after surgery, such as uncontrolled diabetes mellitus; (2) alcoholism; (3) local infection or insufficient bone (on CBCT evaluation) to place an immediate implant; (4) previous bone grafting procedures in the study area; (5) pregnancy; (6) history of radiation therapy to the head or neck region; (7) history of chemotherapy within 5 years prior to surgery; and (8) patients smoking more than 10 cigarettes a day or with drug/alcohol abuse. Patients were informed about the characteristics of the study and signed the corresponding consent. Treatment consisted of three stages (Table 1).

### 2.1. Treatment Stages

#### Diagnostic Phase

**Figure 1:**
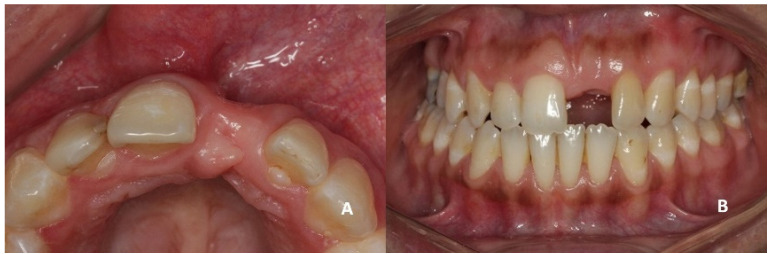
(**A**,**B**) Initial presentation of a patient wearing a removable partial prosthesis replacing a missing left upper central incisor.

On clinical inspection of the aesthetic area, it was observed in all patients that the integrity of the vestibular plate was compromised by significant physiological and structural changes after extraction (Figure 1). This was verified through periapical radiographs and/or panoramic and dental CBCT, which provided sagittal and frontal slices for analysis. On sagittal CBCT images, the anteroposterior distance of the CNP at the level of its upper, middle, and lower thirds was calculated. Axial plane images were in turn used to determine NPC height from the level of the upper bifurcation to the palatal opening, and the width of the residual crest in the toothless zone [24,25].

**Figure 2:**
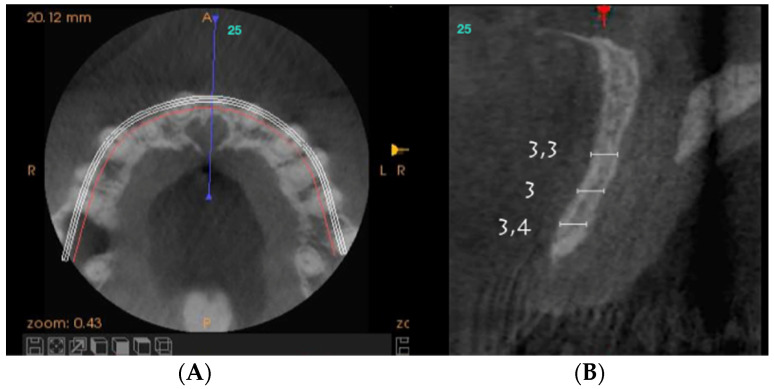
Representative sections of a Cone Beam Computed Tomography (CBCT) scan in which the bone thickness of the maxillofacial region and the widening of the incisive foramen are observed. (**A**) Occlusal view. (**B**) Sagittal view.

Axial sections corresponding to the middle part of the NPC showed the collapse of the vestibular plate and revealed the conduit’s size. All the cases presented thinning of the palatal ridge due to marked widening of the incisive foramen (Figure 2); so, according to the SAC Classification (i.e., simple, advanced, or complex), they were categorized as complex [2]. In addition, careful consideration had to be given to the nasopalatine bundle for correct three-dimensional positioning of the implant. Since the vertical and horizontal defects demanded bone augmentation before implant placement [17], a diagnostic wax-up was performed to determine the amount of bone needed in each case.

According to surgical protocol type IV [17] and due to the time elapsed since dental extraction, volume and height modifications in bone and soft tissue architecture (class IV D [21]), as well as the absence of proximal papillae, were detected in all cases. The loss of biological space was verified in radiographic images and with a periodontal probe. Attending to the above considerations, we planned to place the implant in a correct 3D position, guided by the prosthetic restoration.

### 2.2. Surgical Procedures

Initial studies on the selected cases showed four-wall bone defects [26] involving widening of the incisive foramen, which determined the need for bone augmentation in the horizontal and vertical direction of the ridge. We selected the mandibular symphysis as the donor bone area, because of both its accessibility and its cortico-cancellous structure. 

In all patients, the surgical procedure began with an intrasulcular incision and a papillae base incision on the anteroinferior teeth, from canine to canine (4.3 to 3.3). Auxiliary incisions were next made on distal aspects over the same teeth. The flap design took into account the emergence of the right and left mentonian nerves. 

**Figure 3:**
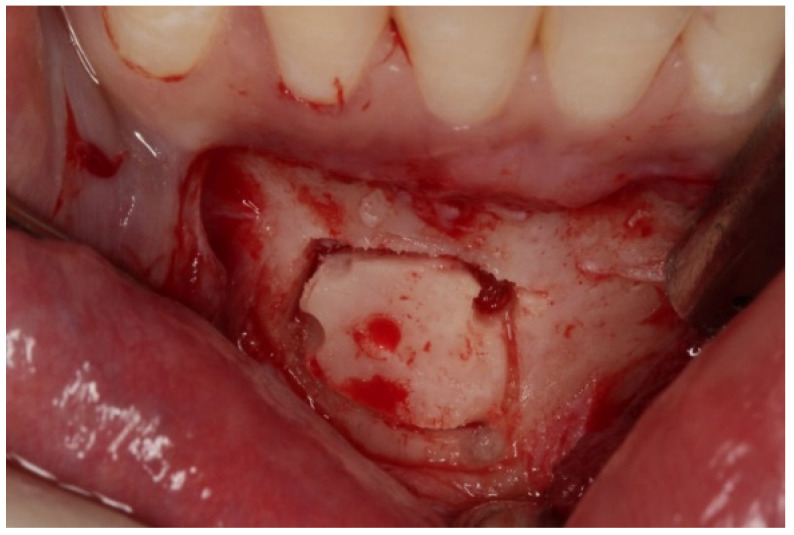
Donor site of the mandible and chin block bone harvesting.

As for the bone block, it was based on the 5 mm rule, which instructs to leave at least 5 mm of bone tissue between the cut and surrounding anatomical landmarks, i.e., root apices of teeth 4.3 to 3.3, mental foramen, and mandibular basal bone [27,28]. The bone block was obtained with a piezoelectric scalpel (Piezosurgery^®^, Mectron^®^ Medical, Carasco, Italy), because it allows exact cuts in hard tissues and its micro-vibrations do not damage adjacent tissues (Figure 3).

**Figure 4:**
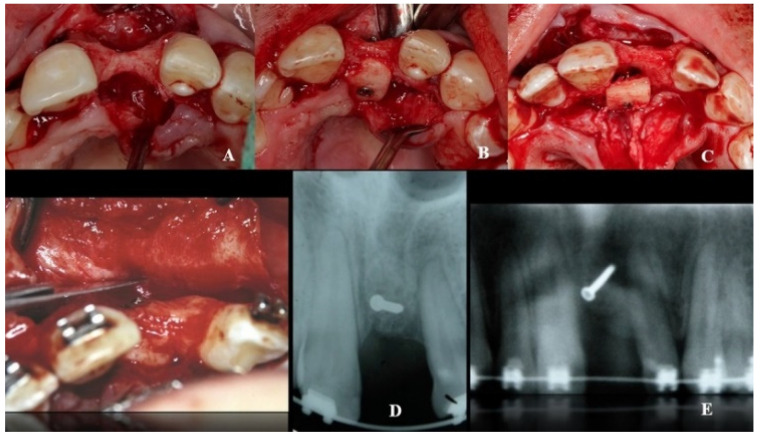
Recipient area (**A**). Location of the bone block at the level of the foramen incisive (**B**) and on its incisal aspect (**C**). (**D**,**E**) Block fixation using fixing screws.

A double approach was used on the recipient jaw. On its vestibular aspect, compensating incisions were made distally on the lateral incisors (1.2 and 2.2), joined by an intrasulcular incision at the level of teeth 1.2 to 2.2, including a central incisor. A crestal incision was traced over the toothless residual ridge. A full-thickness trapezoidal flap was obtained upon curettage. On the palatal side, extensive curettage was performed to obtain a pocket covering the area from 1.2 to 2.2, thus exposing the bone defect and revealing the neurovascular bundle. The latter was next emptied with a bone scraper, and a 20 mm wide × 10 mm high cortico-cancellous block was obtained and placed palatally, covering the incisor foramen to increase the vestibulo–palatal width of the residual ridge. The blocks were held in position by titanium fixation micro-screws (BoneScrew kit^®^ BioHorizons, Birmingham AL, USA) (Figure 4).

Combined application of autogenous bone and a xenograft (BioOss, Geistlich-Pharma AG, Schlieren, Switzerland) was then performed on the palatal, crestal, and vestibular aspects of the defect and canal areas, in order to increase the contour. A resorbable type I collagen membrane (Mem-Lok^®^ RCM, BioHorizons, Birmingham, AL, USA) was then placed on the site, folded to cover the enlarged area, and attached with additional titanium tacks on the buccal side. 

Reconstitution of the donor site was done by layers. The deep layers were sutured with polyglycolic acid (resorbable synthetic suture). First, inverted suturing was used on the periosteal and deep muscular layers to avoid mentonian ptosis of the lower lip and exposure of the lower incisors. Subsequently, the mucosal layer was sutured with a continuous suture. A suspensory suture was next performed in the interdental spaces and auxiliary, simple stitches were applied using non-absorbable 5.0 monofilament nylon. In the recipient area and after the membrane was fixed, the flap was mobilized to allow a tension-free primary closure using periosteum release incisions. 

**Figure 5:**
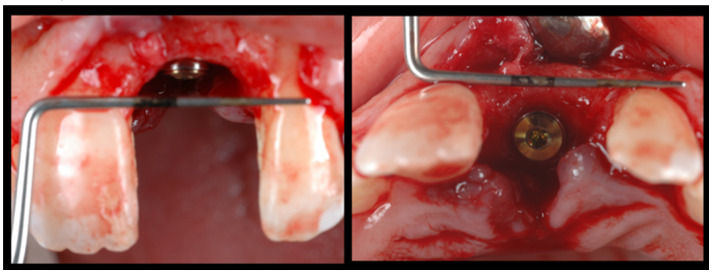
With the help of a surgical guide, implants were positioned in the correct 3D position.

During the second surgery, 6 to 10 months thereafter, the fixation screws were removed. With the help of a surgical guide, Tapered Internal Laser-Lok^®^ 3.8 implants with a 3.5 mm prosthetic platform (BioHorizons, Birmingham, AL, USA) were positioned in the correct 3D position. Provisional restoration consisted of an acrylic crown attached to the teeth adjacent to the gap with an adhesive system (Figure 5). 

### 2.3. Prosthetic Procedures

**Figure 6:**
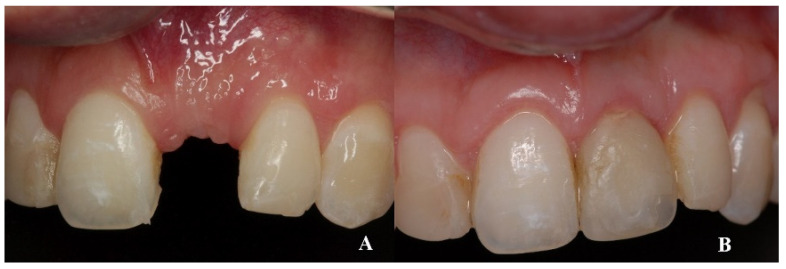
(**A**) Initial provisionalization (adhesive bridge). (**B**) Subsequent customized provisionalization, screwed down to create the emergence profile.

Implants were activated 6 to 10 months after placement. A temporary acrylic crown was screwed to the implant, and a temporary resin abutment (Peek^®^ BioHorizons, Birmingham, AL, USA) was used to shape the cervical contour and emergence profile (Figure 6).

**Figure 7:**
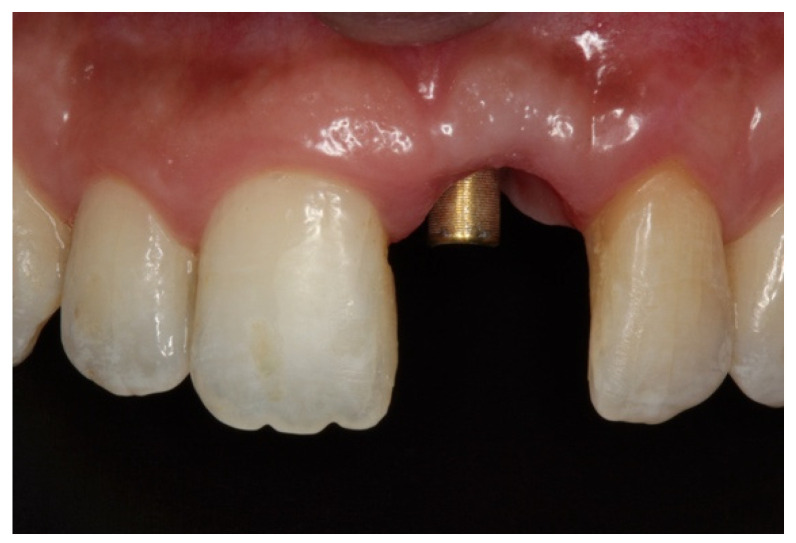
Prosthetic stage. Verification of the Zc coping on the abutment (Ti-Base^®^ BioHorizons Birmingham, AL, USA).

Three months after implant activation, a screw-retained, pure porcelain crown was designed for each patient to optimize aesthetic results. The process began with the transfer of the emergence profile established with the provisional restoration. Models of the maxilla and antagonist teeth were obtained. A Ti-Base^®^ abutment (BioHorizons, Birmingham, AL, USA) was then connected and laser treatment was used for the area of high aesthetic compromise (Figure 7). 

**Figure 8:**
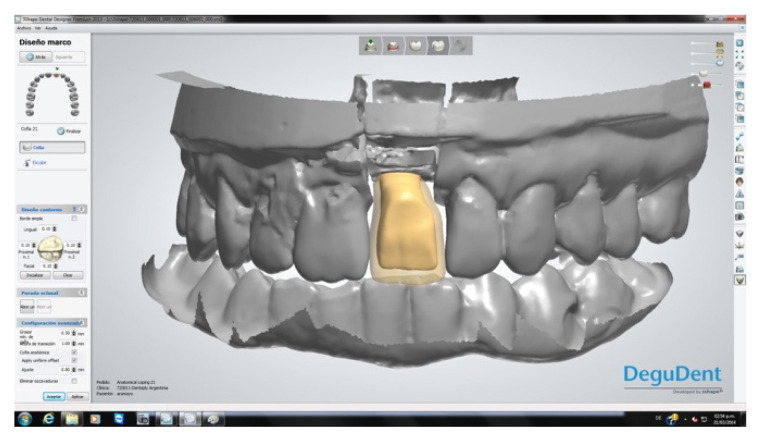
CAD-CAM restoration design.

**Figure 9:**
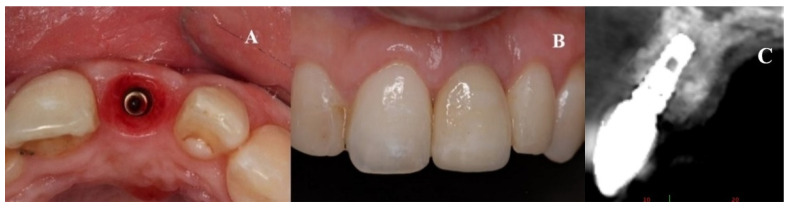
(**A–C**) Placement of the porcelain crown.

**Figure 10:**
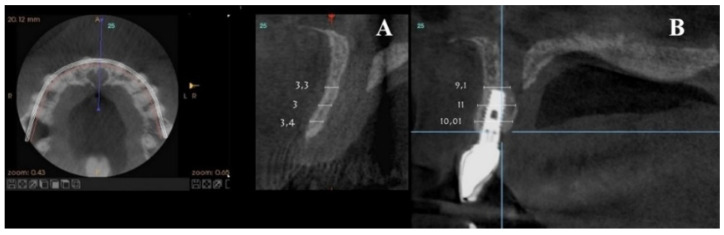
Radiographic control of Patient 1 at their 9-year follow-up. An occlusal view is shown on the left. (**A**) Sagittal view of the nasopalatine duct prior to treatment. (**B**) Post-treatment control radiograph: sagittal view of the implant in a three-dimensional position with the regenerated palatal wall.

The crowns were fabricated using computer-aided design/computer-aided manufacturing (CAD-CAM, Cercon^®^ Dentsply Sirona System, York, PA, USA; Figure 8). After a mock-up to guide color determination, the mesostructure was layered with ceramic having a thermal expansion coefficient compatible with the zirconia in the mesostructure, to produce the crown. The titanium base of the abutment was then joined to the internal surface of the CAD-CAM component by applying Single Bond Universal adhesive^®^ (3M Espe, St. Paul, MN, USA) plus RelyX Unicem^®^ cement (3M Espe, St. Paul, MN, USA) on both surfaces, to transform the system into a single component. Through this procedure, a screw-down crown was obtained, as outlined in the treatment plan. Finally, each crown was screwed applying a torque of 32 N/cm and the palatal access was sealed with a composite of the same color as the porcelain (Figure 9). All patients followed a maintenance program (Figure 10).

### 2.4. Parameters Evaluated

#### 2.4.1. Bone Ridge Thickness

For each patient, pre- and post-treatment CBCT measurements of the anterior ridge thickness of the aforementioned foramen were taken at different heights, i.e., 4, 8, and 14 mm apical to the marginal bone ridge. CBTC was performed at 90 Kv and 10 mA, with an 18-second exposure cycle on a Promax-3D plus equipment (Planmeca, Finland). The obtained images were analyzed with Romexis 4.4.0.R. A field of view of 40 mm by 50 mm was used, with an isoropic voxel size of 75 µm (0.075 mm). Serial slices (0.5 mm in thickness) with 0.5 mm reconstruction intervals were analyzed.

For the scanning procedure, the occlusal planes were oriented parallel to the horizontal plane. Medical imaging recording protocols were used for comparative measurements. First, image relationships were determined following the process of superimposition of tomographic images obtained by CBCT by using the Canny edge detection module of MATLAB R2018a (64-bit). Overlays were made in the areas of interest, obtaining identical reference points as those provided by the Canny filter.

For each patient, pre-surgical measurements were taken in the vestibular table observed from the sagittal plane, from the vestibular cortex to the anterior cortical wall of the NPC. In the same fashion, the vestibulo–palatine width was measured between 6.5 and 9.5 months (7 months on average) after implant activation.

#### 2.4.2. Neurosensory Test

Neurosensory testing was performed when patients returned to have the permanent crown fitted. To this end, the surface of the buccal mucosa was gently touched with a blunt needle to verify any change in sensitivity. This was evaluated in the palatine fibromucosa of the canine and incisor region and classified as normal, hypersensitive, hyposensitive, or anesthetized, as indicated by the patient.

#### 2.4.3. Aesthetic Index

At the end of the follow-up (2 to 9 years), pink and white esthetic scores (PES/WES) [29,30] were determined in five 2–4.5 years-old restorations and in five 6–9 years-old restorations by comparing them with homologous teeth. Four observers (from different specialties: a prosthodontist, a surgeon, a periodontist, and an implantologist) independently assigned a score of 0, 1, or 2 to the following parameters: mesial and distal papilla, curvature of the vestibular mucosa, vestibular mucosa level and convexity of the root/color of the mucosa, and texture of the peri-implant tissue. For WES, scores of 0, 1, or 2 were assigned to the following items: tooth shape and volume, color, surface texture, and translucency. The maximum score for each index was 10.

#### 2.4.4. Treatment Perception

One month after the end of each treatment, patients filled out a validated treatment evaluation questionnaire [31] that addressed whether the treatment was unbearable or comfortably bearable, whether the patient experienced a foreign body sensation in the anterior palatal area, and whether their expectations were met. The questionnaires were analyzed according to published guidelines [32] related to a test technique for measuring subjective or behavioral phenomena (Table 2).

### 2.5. Statistical Analysis 

All data were first analyzed descriptively. Student’s *t* distribution analysis was performed as described by Brunner [33]. The neurosensory test and the patient perception questionnaire were analyzed by frequency statistics, with dichotomous (YES/NO) variables. All statistical tests were performed using R 3.4.2 for Windows (Institute for Statistics and Mathematics, Wirtschafts University, Vienna, Austria). 

## 3. Results

Ten implants were placed in a total of 10 patients with a mean age of 30.5 years (range 25 to 50 years) who presented an anterosuperior toothless gap at the level of the right or left central incisors (1.1 or 2.1). Six implants (67%) were placed in the right central incisor and 4 (33%) in the left central incisor (Table 3).

Pre-surgical vestibulo–palatine width (mean ± standard deviation) of the anterior ridge at different heights (4, 8, and 14 mm apical to the marginal bone ridge) was 3.5 ± 2.0 mm; 5.4 ± 1.5 mm, and 6.1 ± 1.9 mm, respectively. Post-treatment total vestibulo–palatine width was 10.1 ± 2.0 mm, 10.5 ± 1 mm, and 13.4 ± 3.0 mm, respectively (Table 4). 

Post-treatment, results of the questionnaire on patient perception showed that all patients (100%) deemed treatment “comfortably bearable”, none (0%) experienced a foreign body sensation in the anterior sector of the palate, and all patients (100%) indicated that they were “completely satisfied” with the treatment received. The neurosensory test yielded the following results: normal, 100%; hypersensitive, 0%; hyposensitive, 0%; anesthetized, 0%.

At the end of the follow-up of each restoration/patient, PES and WES were measured. The assessment facilitated an objective appreciation of the crown and peri-implant tissue with a maximum score of 20 points and an acceptable minimum of six points. Mean PES and WES of 7.5 and 7.0, respectively, were recorded (Table 5). No biological or technical complications arose in any patient during the follow-up period.

## 4. Discussion 

The relationship of the anterior toothless gap with the NPC is important in the rehabilitation of the esthetic zone, especially when there are anatomical variations in the recipient zone of the implant. Long-term studies on outcomes of bone augmentation procedures at the NPC level are scarce. Here, correct positioning of the implants and aesthetics demands are more challenging due to the presence of the homologous counterpart.

Buser et al. [24] advocated late implant placement (type 4) in order to optimize results and reduce morbidity and treatment times. For this purpose, contour augmentation is performed with autogenous bone particles collected locally, in order to accelerate the rate of new bone formation. A non-reticulated, resorbable collagen membrane is used to avoid removing the membrane on a second surgery. In the present research work, in each patient, a combination of autogenous bone and a xenograft was used to increase the contour in the region of the defect and the conduit, on its palatal, crestal, and vestibular aspect as well. A type I collagen membrane was placed.

The effectiveness of early implant placement with simultaneous augmentation of the contour through GBR was studied by Chappuis et al. [25], with two layers of composite graft placed in aesthetic sites of a single post-extraction tooth in 20 patients with a 10-year follow-up. The median peri-implant bone loss was 0.35 mm between 1 and 10 years, with a 95% success rate, good esthetic results, and a high median PES of 8. In our investigation, the aesthetic results also met our expectations (mean PES and WES of 7.5 and 7.0, respectively).

The work of Cordaro and Terheyden et al. [27] describing a phased approach to horizontal ridge augmentation is also worth noting. In their study, residual ridges of 3.19 mm recorded linear gains of 4.3 mm at implant placement, with complication rates between 2.5% and 10% for graft exposure. Meanwhile, with GBR on residual ridges at least 2.9 mm wide, they recorded an average gain of 3.31 mm and a complication rate of 15% for membrane exposure. In the cases treated by our research team, there were no graft or membrane exposures.

Peñarrocha et al. [34] studied seven edentulous patients with severe bone resorption for three to seven years (mean of five years). They received seven implants in the nasopalatine buttress and 29 implants in its posterior area. At the end of the observation period, the patients were satisfied with the comfort and stability of the prostheses and manifested no sensory alterations. The authors concluded that implant placement in the NPC region can be a viable treatment approach for the rehabilitation of the severely atrophied maxilla. In the present work, a block graft plus a bone biomaterial was placed palatally, as patients had widening of the incisive foramen, suggesting that the therapy under evaluation may be appropriate for this situation.

Bornstein et al. [4] studied the dimensions and anatomical characteristics of the NPC. The research involved 44 men and 56 women and 100 CBCT scans obtained between 2007 and 2009 were analyzed. According to NPC morphology, i.e., single, double parallel, and Y-shaped, with variations (two or more Stenson’s foramina), 45% of individuals had a single NPC, 15% had two separated, parallel ducts, and 40% showed Y-type variations. Dimensional analysis of the NPC revealed a mean diameter of 3.49 mm in its nasal opening and a clearly wider incisor foramen in men, with a mean diameter of 4.45 mm. The length of the NPC was 10.99 mm and was also significantly longer in males. The buccal plate showed an increasing width from the bone crest toward the apical area, with mean values of 6.5 mm, 6.59 mm, and 7.6 mm in the crestal, middle, and apical regions, respectively. The time elapsed since the loss of the central incisors also had a significant influence on the vestibular width of the ridge.

Almache et al. [35] compared tomography scans of the anterior maxilla from dentate and edentulous patients and found that toothed patients have higher maxillary heights. In turn, lower-, middle-, and upper-level diameter measurements at NPC cortices revealed smaller distances in toothed patients, while transversal enlargement at the level of the NPC characterized edentulous ones. It was concluded that in the study population (patients aged 40 to 70 years, of both sexes) topographic structural variations (greater width, shorter length, greater transverse dimension, and overall NPC enlargement) distinguish individuals with anterior maxillary edentulism from non-edentulous ones. In our edentulous cases, CT scans evidenced NPC widening, especially at the level of the incisive foramen. In light of the evidence above, this could be ascribed to the edentulous condition, which clearly impacts NPC stability and morphology.

Our work is also in agreement with a two- to four-year retrospective study carried out by Belser et al. [31] that involved 45 patients in which the anterior sector of the maxilla was treated with a single implant in accordance with the concept of early implant placement. The study demonstrated that the placement of a unitary implant is a predictable treatment with a high probability of success. The index used confirmed an objective evaluation of the result, and a mean score of ~7 was registered for both PES/WES indexes in soft and hard tissues. The prosthetic restoration performed was integrated into the system in harmony with the existing white and pink aesthetics, consisting of an optimal biomimetic approach.

In a systematic review of implant loading and placement protocols, Gallucci et al. [21] found that out of 898 implants placed with protocol type 4C (late implant placement and conventional loading) only 11 failed. The weighted cumulative survival rate was 97.7% with a mean of 30.6 months follow-up, and the success rate ranged from 88% to 100%. The type 4C was among the most scientifically documented and clinically validated protocols, particularly when treatment is modified with bone augmentation, low insertion torque, involves implants with reduced diameter, and is performed in patients with local and systemic factors.

In our tomographic studies, a marked ridge collapse was generally observed. The NPC has been proposed as an anatomical buttress suitable for implant placement, and the technique used was previously described by Resnik [36]. The anterior support provided by a nasopalatine implant decreases the bending moment created in the vertical plane when the prosthetic restoration is placed, which improves biomechanics when selecting either a class I occlusal relationship, or an edge-to-edge or inverse articulation. All this contributed to the conditioning of the area so that each implant is in the proper three-dimensional position with its prosthetic rehabilitation.

In the 10-year retrospective study by Urban et al. [37], 20 patients were treated with implant-supported fixed restorations. It was concluded that regeneration of bone defects in the anterior maxillary area with augmentation of the bone crest in the horizontal and/or vertical direction, including lateralization of the nasopalatine nerve and implant placement in a second surgery, represents a predictable surgical technique. Meanwhile, in a prospective cohort study, Ewers [38] placed short implants in the atrophic maxillae of nine patients during the observation period. No implants were lost, and the patients did not experience any sensory disturbance due to implant placement in the NPC. 

In our study a different technique than Urban’s [37] was used, since rather than lateralizing the neurovascular package, this was in all cases emptied of nervous and vascular structures in the same graft surgery. Once healed, this area allowed placing the implant in the planned 3D position. 

In the work performed by Wasdrop [22], the neurovascular bundle was removed and an allogeneic block graft was placed in the NPC. The block was placed vestibularly and the membrane used extended from the vestibular to the palatal regions. Our approach was different, as we removed the neurovascular bundle over the palatine aspect without altering the walls of the duct. This protected the scarce vestibular material remaining, which functioned as an anchorage point for the bone graft that was placed vestibularly to fill and improve the facial appearance.

Our study also agrees with that of Tözüm et al. [39], who studied 933 partially edentulous patients and demonstrated that the length of the canal was shortened in the edentulous anterior maxilla compared to the dentated maxilla, although without significant differences in canal diameter between groups. They further showed that the NPC was longer and wider in males, that the shape of the canal was cylindrical in ~41% of images, and that the diameter of the NPC does not increase with the length of the duct nor with age. Unlike our study, which mainly focused on the vestibular bone crest due to its relevance to patient aesthetics, Tözüm et al. addressed the palatine bone crest.

Cavallaro et al. [23] reviewed the literature for human clinical reports addressing implant placement at or near the NPC. They determined that although considerable variation exists, the NPC is generally about 10 mm long and 4 mm wide and tilts from the horizontal plane at an angle of 66 degrees. Several clinical reports demonstrate that the conduit can be enucleated and grafted into bone prior to successful implant placement. It is possible to place an implant in the NPC at the time of surgery, and this procedure may be combined with a bone graft. Numerous reports indicate that there is generally no permanent loss of sensation in the anterior sector of the palate when an implant is placed in the NPC. Therefore, the authors concluded that placing an implant in the NPC is a viable procedure as part of a surgical and prosthetic treatment plan in cases of severe atrophy of the jaw, combination syndrome, or those presenting with a large NPC. The comprehensive evidence presented by the referred review [23] provides scientific support to our clinical work, which entailed NPC enucleation and widening of the incisive foramen.

Additional support to our approach is provided by Dos Santos et al. [40], who placed implants in the NPC to aid the rehabilitation of maxillary atrophy. In the first stage, surgery was performed to lift the maxillary sinus membrane and for xenogenic bone grafting. In the second stage, seven implants were placed in the maxilla; one was anchored in the NPC after enucleation using the implant system’s drill threads. After five months, the implant was reopened, followed by molding, fabrication, and placement of the prosthesis. No painful symptoms were recorded over a four-year follow-up. 

Staderini et al. [41] studied whether facial features and observers’ gender affect the aesthetic scores of patients with cleft lip and unilateral cleft palate (UCLP) after soft tissue reconstruction. Although no cleft lip/UCLP cases were addressed in the present work, the mentioned study is interesting regarding the final reconstruction assessment.

The main advantages of our approach lie in the effective recovery of the NPC as a palatal buttress for the rehabilitation of single-tooth gaps after the loss of an upper central incisor. This strategy should be also valuable in the rehabilitation of severely atrophic edentulous sites in which conventional treatments do not allow implant placement due to the defect of the bone remnant. Since this type of bone defect is not fairly frequent, more studies in a larger set of patients should be carried out to reach definitive conclusions.

Noteworthy, in the present study, no post-operative pain was experienced by any patient over a two- to nine-year observation time. All implants were maintained and exhibited adequate osseointegration, none of the patients reported sensory alterations after a six-month follow-up, and acceptable standards for PES/WES aesthetics were achieved in all cases.

## 5. Conclusions 

The surgical technique described for the regeneration of bone defects in the anterior maxilla through horizontal and/or vertical ridge augmentation with NPC involvement prior to implant placement should yield predictable results when strict patient inclusion criteria are applied. The stability of the tissues and the aesthetics results achieved indicates that this regenerative technique is able, in specific clinical cases and through a palatal approach, to correctly position the 3D implant and obtain adequate bone augmentation in the horizontal direction.

The current study involved only 10 implants in an equal number of patients, which were followed over an average period of 3.5 years. Therefore, a larger sample of patients and a longer follow-up period are required to validate the findings of this pilot study.

## Figures and Tables

**Table 1:** Planning guide for rehabilitation of anterior tooth gaps.

Planning Guide for Rehabilitation of Anterior Tooth Gaps	
**Diagnostic Stage**	Clinical evaluation/Gap volume assessment/CBCT analysis/Diagnostic wax-up modeling.
**Surgical Stage**	Site preparation/Bone volume augmentation through autologous chin block graft with rigid fixation, in combination with GBR. After 6–10 months of graft maturation, removal of the fixation screws from the grafted blocks and placement of the implant in a suitable 3D position with submerged healing. Second surgery, after 6–10 months of osseointegration, activation of the implant and placement of a screw-retained provisional to create optimal cervical contour and emergence profile for 3 months.
**Prosthetic Stage**	Transfer of the cervical contour and emergence profile obtained during provisionalization. Use of a Ti-Base abutment for restoration with a screw-down crown of pure (CAD-CAM) or stratified ceramic.

**Table 2:** Patient perception in relation to treatment.

Treatment Perception Questionnaire			
Item	*N*	Yes (%)	No (%)
Treatment was comfortably bearable	10	100	0
Treatment was unbearable	10	0	100
Did you notice a foreign body sensation in the anterior palate?	10	0	100
Were your expectations (regarding the treatment performed) met?	10	100 *^1^	0 *^2^

*^1^: fully satisfied; *^2^: completely unsatisfied.

**Table 3:** Patient characteristics and surgical sites treated with horizontal/vertical augmentation in the nasopalatine canal.

Healing Time and Maintenance							
Patient	Gender	Age	Tooth	Regeneration	Grafting (Months)	Implant (Months)	Load (Years)
1	F	35	2.1	(HA)	8	8	9
2	F	33	1.1	(HA)	7	6	8.5
3	F	38	1.1	(HA)	10	9	7
4	M	50	2.1	(HA)	8.5	7	6
5	F	40	1.1	(HA)	9.5	6	6
6	F	25	2.1	(HA)	7.5	10	4.5
7	M	30	1.1	(HA)	6.5	7.5	3.5
8	F	32	2.1	(HA)	8	8	3
9	F	48	2.1	(HA)	9	10	2.5
10	F	32	2.1	(HA)	8.5	9	2

HA: autologous and particulate block graft; F: female; M: male.

**Table 4:** CBTC-based bone ridge thickness measurements.

Apical Distance to the Marginal Ridge	Vestibulo–Palatine Width of the Anterior Ridge (Pre-Surgery)	Total Vestibulo–Palatine Width (Post-Treatment)	Difference	*p*
4 mm	3.5 ± 2.0 mm	10.0 ± 2.0 mm	6.5 mm	1.7 × 10^−5^
8 mm	5.4 ± 1.5 mm	10.5 ± 1.0 mm	5.1 mm	1.0 × 10^−5^
14 mm	6.1 ± 1.9 mm	13.4 ± 3.0 mm	7.3 mm	6.3 × 10^−5^

Mean ± standard deviation of ridge thickness at the level of the incisive foramen for various heights (4 mm, 8 mm, and 14 mm apical to the marginal bone crest) (*n* = 10). *p*: Student’s *t*-test.

**Table 5:** Pink and white aesthetic scores (PES/WES) at the end of the follow-up (2 to 9 years).

			**PES (*n* = 10)**			
	**Mesial Papilla**	**Distal Papilla**	**Curvature of Vestibular Mucosa**	**Level of Vestibular Mucosa**	**Root Convexity, Mucosa Color,** **and Texture of Peri-Implant Tissue**	**Total**
**Maximum**	2	2	2	2	1	8
**Minimum**	1	1	1	1	0	6
**Mean**	1.6	1.28	1.93	1.8	1.18	7.5
**SD**	0.6	0.51	0.3	0.45	0.5	0.8
			**WES (*n* = 10)**			
	**Tooth Shape**	**Tooth Volume**	**Color**	**Surface Texture**	**Translucency**	**Total**
**Maximum**	2	2	2	2	2	10
**Minimum**	1	1	1	0	1	4
**Mean**	1.2	1.4	1.3	1.7	1.3	7
**SD**	0.5	0.4	0.5	0.5	0.5	1.5

Combined data from implant restorations at 2.0 to 4.5 years (*n* = 5) and 6 to 9 years (*n* = 5) follow-up.SD: Standard deviation. Maximum value of PES = 10 (modification according to Belser et al. [24]).
